# HGF/SF and its receptor c-MET play a minor role in the dissemination of human B-lymphoma cells in SCID mice

**DOI:** 10.1038/sj.bjc.6690649

**Published:** 1999-09

**Authors:** I S Weimar, K Weijer, P C M van den Berk, E J Muller, N Miranda, A Q Bakker, M H M Heemskerk, A Hekman, G C de Gast, W R Gerritsen

**Affiliations:** Division of Immunology, Netherlands Cancer Institute, Plesmanlaan 121, Amsterdam, 1066 CX, The Netherlands; European Cancer Center, Amsterdam, The Netherlands; Department of Medical Oncology, Netherlands Cancer Institute, Plesmanlaan 121, Amsterdam, 1066 CX, The Netherlands

**Keywords:** HGF/SF, c-MET, retroviral transduction, human B-lymphoma cells, dissemination, SCID mice

## Abstract

The MET protooncogene, c-MET, encodes a cell surface tyrosine kinase receptor. The ligand for c-MET is hepatocyte growth factor (HGF), also known as scatter factor (SF), which is known to affect proliferation and motility of primarily epithelial cells. Recently, HGF/SF was also shown to affect haemopoiesis. Studies with epithelial and transfected NIH3T3 cells indicated that the HGF/SF–c-MET interaction promotes invasion in vitro and in vivo. We previously demonstrated that HGF/SF induces adhesion of c-MET-positive B-lymphoma cells to extracellular matrix molecules, and promoted migration and invasion in in vitro assays. Here, the effect of HGF/SF on tumorigenicity of c-MET-positive and c-MET-negative human B-lymphoma cell lines was studied in C.B-17 *scid/scid* (severe combined immune deficient) mice. Intravenously (i.v.) injected c-MET-positive (BJAB) as well as c-MET-negative (Daudi and Ramos cells) B-lymphoma cells formed tumours in SCID mice. The B-lymphoma cells invaded different organs, such as liver, kidney, lymph nodes, lung, gonads and the central nervous system. We assessed the effect of human HGF/SF on the dissemination of the B-lymphoma cells and found that administration of 5 μg HGF/SF to mice, injected (i.v.) with c-MET-positive lymphoma cells, significantly (*P* = 0.018) increased the number of metastases in lung, liver and lymph nodes. In addition, HGF/SF did not significantly influence dissemination of c-MET-negative lymphoma cells (*P* = 0.350 with Daudi cells and *P* = 0.353 with Ramos cells). Thus the effect of administration of HGF/SF on invasion of lymphoma cells is not an indirect one, e.g. via an effect on endothelial cells. Finally, we investigated the effect of HGF/SF on dissemination of c-MET-transduced Ramos cells. In response to HGF/SF, c-MET-transduced Ramos cells showed an increased migration through Matrigel in Boyden chambers compared to wild-type and control-transduced Ramos cells. The dissemination pattern of c-MET-transduced cells did not differ from control cells in in vivo experiments using SCID mice. Also no effect of HGF/SF administration could be documented, in contrast to the in vitro experiments. From our experiments can be concluded that the HGF/SF–c-MET interaction only plays a minor role in the dissemination of human B-lymphoma cells. © 1999 Cancer Research Campaign


*British Journal of Cancer* (1999) **81**(1), 43–53
© 1999 Cancer Research Campaign
Article no. bjoc.1999.0649

### HGF/SF and its receptor c-MET play a minor role in the dissemination of human B-lymphoma cells in SCID mice

**IS Weimar[1], K Weijer[1], PCM van den Berk[1], EJ Muller[1], N Miranda[1,2], AQ Bakker[1], MHM Heemskerk[1], A Hekman[1], GC de Gast[1,3] and WR Gerritsen[1,3]***

[1]Division of Immunology, Netherlands Cancer Institute, Plesmanlaan 121, 1066 CX Amsterdam, The Netherlands; [2]European Cancer Center, Amsterdam, The Netherlands; [3]Department of Medical Oncology, Netherlands Cancer Institute, Plesmanlaan 121, 1066 CX Amsterdam, The Netherlands

**Summary** The MET protooncogene, c-MET, encodes a cell surface tyrosine kinase receptor. The ligand for c-MET is hepatocyte growth factor (HGF), also known as scatter factor (SF), which is known to affect proliferation and motility of primarily epithelial cells. Recently, HGF/SF was also shown to affect haemopoiesis. Studies with epithelial and transfected NIH3T3 cells indicated that the HGF/SF–c-MET interaction promotes invasion in vitro and in vivo. We previously demonstrated that HGF/SF induces adhesion of c-MET-positive B-lymphoma cells to extracellular matrix molecules, and promoted migration and invasion in in vitro assays. Here, the effect of HGF/SF on tumorigenicity of c-MET-positive and c-MET-negative human B-lymphoma cell lines was studied in C.B-17 *scid/scid* (severe combined immune deficient) mice. Intravenously (i.v.) injected c-MET-positive (BJAB) as well as c-MET-negative (Daudi and Ramos cells) B-lymphoma cells formed tumours in SCID mice. The B-lymphoma cells invaded different organs, such as liver, kidney, lymph nodes, lung, gonads and the central nervous system. We assessed the effect of human HGF/SF on the dissemination of the B-lymphoma cells and found that administration of 5 μg HGF/SF to mice, injected (i.v.) with c-MET-positive lymphoma cells, significantly ($P$ = 0.018) increased the number of metastases in lung, liver and lymph nodes. In addition, HGF/SF did not significantly influence dissemination of c-MET-negative lymphoma cells ($P$ = 0.350 with Daudi cells and $P$ = 0.353 with Ramos cells). Thus the effect of administration of HGF/SF on invasion of lymphoma cells is not an indirect one, e.g. via an effect on endothelial cells. Finally, we investigated the effect of HGF/SF on dissemination of c-MET-transduced Ramos cells. In response to HGF/SF, c-MET-transduced Ramos cells showed an increased migration through Matrigel in Boyden chambers compared to wild-type and control-transduced Ramos cells. The dissemination pattern of c-MET-transduced cells did not differ from control cells in in vivo experiments using SCID mice. Also no effect of HGF/SF administration could be documented, in contrast to the in vitro experiments. From our experiments can be concluded that the HGF/SF–c-MET interaction only plays a minor role in the dissemination of human B-lymphoma cells.

**Keywords:** HGF/SF; c-MET; retroviral transduction; human B-lymphoma cells; dissemination; SCID mice

Disseminating lymphoma cells have to adhere to and cross blood vessel walls and the underlying matrices of the tissues in which metastases are formed. Migration of lymphoma cells is thought to be a multistep process similar to what has been described for migration of leucocytes at places of inflammation (Tavassali and Hardley, 1990; Springer, 1994). For transendothelial migration, different adhesion molecules are important, such as selectins, CD44 and integrins (Lawrence and Springer, 1991; Carlos and Harlan, 1994; Zannettino et al, 1995; DeGrendele, 1996). The latter have to be activated by chemoattractants, which are bound to endothelial cells. Chemoattractants are important in activating integrins and in directing the migration of the leucocytes (chemotaxis; Berman and Muller, 1996; Butcher and Picker, 1996; Wakelin et al, 1996). Examples of chemoattractants are platelet-activating factor (PAF), the complement product C5a, interleukin-8 (IL-8) and MIP1α (Baggiolini et al, 1994; Strieter et al, 1996; Adams and Lloyd, 1997). Recently, hepatocyte growth factor (HGF), also known as scatter factor (SF), was identified as a chemoattractant for a subset of T-lymphocytes (Adams et al, 1994). HGF/SF is produced by mesenchymal cells and was first identified as a major mediator of liver regeneration (Michalopoulos and Zarnegar, 1989; Nakamura et al, 1989). It also has mitogenic, morphogenic and motogenic effects on epithelial cells (e.g. mammary, kidney, intestinal and bronchial epithelial cells) as well as on endothelial cells (Stoker et al, 1987; Gerardi and Stoker, 1990; Michalopoulos, 1990; Bussolino et al, 1992; Rong et al, 1992, 1993, 1994; Giordano et al, 1993; Grant et al, 1993; Rubin et al, 1993; Bellusci et al, 1994; Dignass et al, 1994). Recently, a role has been assigned to HGF/SF in hemopoiesis, since HGF/SF was found to affect proliferation, adhesion and survival of haemopoietic progenitor cells (Kmiecik et al, 1992; Mizuno et al, 1993; Galimi et al, 1994; Nishino et al, 1995; Zarnegar and Michalopoulos, 1995; Goff et al, 1996, 1997; Weimar et al, 1998). The receptor for HGF/SF is encoded by the MET proto-oncogene, c-MET, which is a cell surface tyrosine kinase receptor consisting of an extracellular α- and a transmembrane β-chain with the tyrosine kinase domain (Giordano et al, 1989; Naldini et al, 1991*a*, 1991*b*).

Previous results obtained in our laboratory demonstrated that c-MET is expressed in either early or activated normal human B-cells as well as in lymph node samples from non-Hodgkin's lymphoma and Hodgkin's disease patients. c-MET expression was also determined in some human B-lymphoma cell lines (two out of

*Received 8 September 1998*
*Revised 9 March 1999*
*Accepted 10 March 1999*

*Correspondence to:* WR Gerritsen, Department of Medical Oncology, Academic Hospital Free University, Boelelaan 1117, 1007 MB Amsterdam, The Netherlands five cell lines tested were c-MET-positive). Furthermore, we found that HGF/SF induced adhesion of c-MET-positive (but not of c-MET-negative) human B-lymphoma cells to the extracellular matrix (ECM) molecules fibronectin and collagen. Adhesion appeared to be mediated by the integrins $\alpha_4\beta_1$ and $\alpha_5\beta_1$. In addition, HGF/SF promoted migration of c-MET-posit (and not of c-MET-negative) human B-lymphoma cells through Matrigel in Boyden chambers and invasion in rat fibroblast monolayers (Weimar et al, 1997). These results suggest that HGF/SF-c-MET interaction may promote dissemination of lymphoma cells in vivo. Here, we investigated the tumorigenicity of both c-MET-positive and c-MET-negative human B-lymphoma cells in severe combined immune deficient (SCID) mice, with and without administration of human HGF/SF. Human HGF/SF significantly promoted dissemination of c-MET-positive lymphoma cells (BJAB cells) and did not affect dissemination of c-MET-negative tumour cells (Ramos cells). To test the hypothesis that HGF/SF promotes the dissemination of c-MET-positive B-lymphoma cells, we transduced c-MET-negative B-cells (Ramos) with the c-MET gene and studied their capacity to migrate in vitro and in vivo. C-MET transduction resulted in an increased migration of c-MET-transduced Ramos cells through Matrigel in response to HGF/SF, whereas HGF/SF did not affect migration of wild-type and control-transduced cells. The dissemination pattern of c-MET-transduced, control-transduced, or wild-type Ramos cells was investigated in vivo by injecting the cells in SCID mice.

### MATERIALS AND METHODS

### Cells

B-cell lines (BJAB, Raji, Ramos and Daudi) and NIH3T3 cells were obtained from the ATCC (Rockville, MD, USA). The c-MET-transfected NIH3T3 cell line was a generous gift from Prof. GF Vande Woude (National Cancer Institute, Frederick, MD, USA). All cell lines were cultured in Iscove's modified Dulbecco's medium (IMDM; Gibco-BRL, Gaithersburg, MD, USA), supplemented with 5% heat inactivated fetal calf serum (FCS; BioWittaker, Brussels, Belgium), penicillin (100 U $ml^{-1}$; Gibco-BRL) and streptomycin (100 μg $ml^{-1}$; Gibco-BRL).

### Tumorigenicity establishment of B-lymphoma cells in SCID mice

C.B-17 *scid/scid* (SCID) mice were bred and maintained at the Animal Department of The Netherlands Cancer Institute. The mice were kept in isolators under specific pathogen-free conditions and used when 6–10 weeks old. All experiments were approved by the Animal Experimental Advisory Board of The Netherlands Cancer Institute.

Mice were injected intravenously (i.v.; in the tail vein) with $5 \times 10^6$ B-cell tumour cells. In the first pilot experiment HGF/SF (5 μg per mouse) was obtained from R&D Systems, Abingdon, UK (produced in Sf21 insect cells) and was mixed with the tumour cells before i.v. injection, while in all other in vivo experiments, HGF/SF was produced in our laboratory (see below), and was injected subcutaneously (s.c.; into the right flank; 5 μg (or in one experiment 25 μg) per mouse per day; 7 ×; starting on the day of the i.v. tumour injection).

After 3–5 weeks, mice appeared ill (signs of paralysis, ovarian tumour, loss of weight, etc.) and all mice in the same experiment were sacrificed and examined. Organs were fixed (in 4% formaldehyde), embedded in paraffin after which sections were stained for human B-leucocytes (monoclonal mouse anti-human leucocyte common antigen [LCA], No M701, Dakopats, Glostrup, Denmark).

### HGF/SF production

HGF/SF was produced by *Spodoptera frugiperda* 9 (Sf9) insect cells using the baculo-gold-virus-producing system as described previously (Yee et al, 1993; HGF/SF cDNA was a gift from Prof T Nakamura). In short, Sf9 insect cells were infected with baculovirus containing the HGF/SF gene, and secreted (±2 μg $ml^{-1}$ as measured by enzyme-linked immunosorbent assay (ELISA) biologically active HGF/SF (tested in proliferation assays and colony assays) with human bone marrow cells on which HGF/SF acts as a synergistic proliferative factor with other growth factors (Weimar, 1998), tested in a proliferation assay with c-MET-transfected NIH3T3 cells; tested in a scattering assay with MDCK cells (Hordijk et al, 1997). HGF/SF in supernatant of the Sf9 cells was purified using a heparin column and eluted with 1.5 M sodium chloride. Usually, 5 μg HGF/SF was injected s.c. per mouse per day, which contained 70 ng endotoxin. In some experiments HGF/SF was denaturated (30 min autoclaved) to administer exactly the same amount of endotoxin in both groups (the autoclaved HGF/SF was no longer biologically active as tested in colony assay with human bone marrow cells). Subcutaneous injections of 5 μg HGF/SF daily for 7 days did not affect the mice. No illness or other dysfunctions were observed.

### Proliferation assays

#### *$^3$H-thymidine incorporation assay*

Five thousand cells were plated per well in a 96-well plate (Greiner; flat bottom) in IMDM (Gibco-BRL, Gaithersburg, MD, USA), supplemented with 5% heat inactivated FCS (BioWittaker, Brussels, Belgium), penicillin (100 U $ml^{-1}$; Gibco-BRL) and streptomycin (100 μg $ml^{-1}$; Gibco-BRL). After 1 day, the cells were pulsed for 4 h with $^3$H-thymidine (0.5 μCi per well) and counted in a β-plate counter (1205 betaplate™, Finland).

#### *Colony formation assay*

A total of $1 \times 10^5$ mononuclear human and murine bone marrow cells were suspended in 1 ml of IMDM + L-glutamine (Gibco-BRL), supplemented with 10% FCS (BioWittaker), P/S, $7.3 \times 10^{-5}$ M α-monothioglycerol (Sigma, Zwijndrecht, The Netherlands) and 0.36% agarose (SeaPlaque GTG; FMC, Rockland, ME, USA) and were plated in triplicate in petri dishes (35 × 10 mm Falcon/Becton Dickinson, Plymouth, UK). The number of colonies (a colony is 20 cells or more) were scored after 2 weeks by an inverted microscope.

### HGF/SF ELISA

HGF/SF was determined by a HGF-ELISA kit (Institute of Immunology Co. Ltd, Tokyo, Japan). HGF/SF detection is based upon a sandwich method using a solid phase coated anti-human HGF/SF mouse monoclonal antibody and a peroxidase-labelled anti-human HGF/SF mouse monoclonal antibody. Amounts from 0.1 ng $ml^{-1}$ were reliably detected according to the linearity of the standard curve.

 © 1999 Cancer Research Campaign

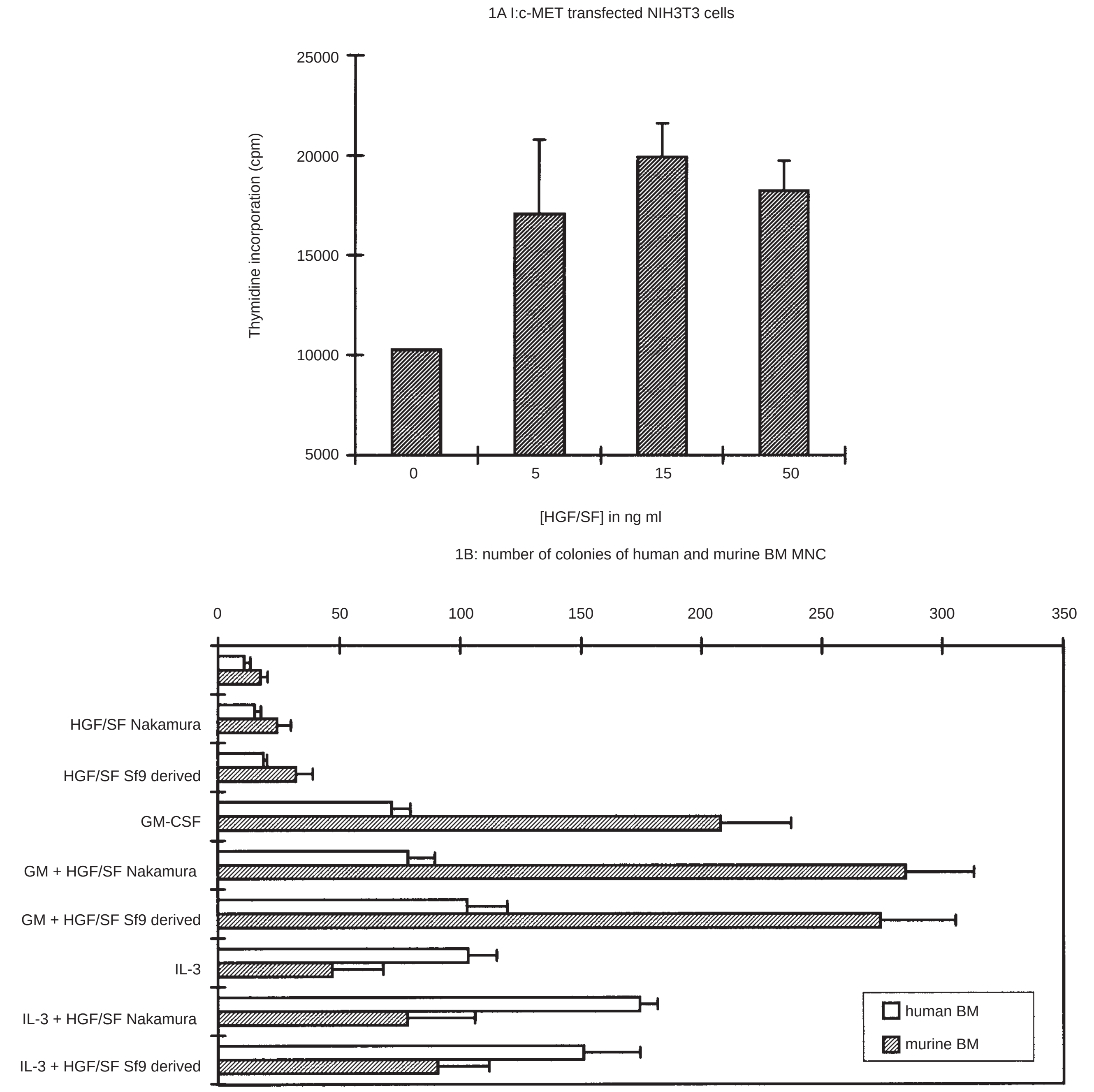

**Figure 1** (**A**) Thymidine incorporation (of 4 hours) of c-MET transfected NIH3T3 cells (**A**-I) and wild-type NIH3T3 cells (**A**-II) with and without HGF/SF (5, 15 and 50 ng $ml^{-1}$). Values are means from triplicates ± s.d. (**B**) Colony formation of $1 \times 10^5$ human and murine mononuclear bone marrow cells in 2 weeks. Values are mean number of colonies ± s.d. from triplicate cultures

### Construction of the c-MET vector and transduction

The c-MET coding sequence was cloned from the pMB1 plasmid (generous gift from Prof. T Nakamura) by polymerase chain reaction (PCR) with the following primers: 5′ CGCGCTCGAGC-CATGGAGGCCCCCGCTGTGCTTGCA; 3′ GATCCCGGGCA-CTATGATGTCTCCCAGAAGG.

The PCR product was cut with *Nco*I (introduced with PCR primer; start codon) and *Swa*I (downstream of the stop-codon), ligated in pSP73 (with *Xho*/EV), and after ± 140 bp exchanged with wild-type cDNA (without PCR; with *Sst*II and *Cla*I). c-MET cDNA (4175 bp) was cut from pSP73 (with *Xho/Sca*I) and ligated between the *Xho*I and the *Sna*BI site of the polylinker from the plasmid LZRS-linker-IRES-EGFP (a modified version of GFP with an enhanced expression (obtained from Clontech Laboratories, Palo Alto, CA, USA) (Heemskerk et al, 1997), to obtain the retroviral vector LZRS-c-MET-IRES-GFP, containing LTR and Ψ packaging sequences from the Moloney murine leukaemia virus (Mo-MuLV) as well as puromycin resistance gene.

**Table 1** Effect of HGF/SF on migration through Matrigel of c-MET-transduced RAMOS cells

| | | Number of migrated cells | |
|---|---|---|---|
| Experiment | RAMOS cells | Without HGF/SF | With HGF/SF |
| 1 | wild-type | 267 | 225 |
| | GFP | 339 | 385 |
| | c-MET/GFP | 301 | 542 |
| 2 | wild-type | 189 | 194 |
| | GFP | 213 | 225 |
| | c-MET/GFP | 202 | 796 |

Ramos cells ($5 \times 10^5$) wild-type, control-transduced (with GFP alone), and c-MET-IRES-GFP-transduced (c-MET/GFP) were mounted in 1.5 ml culture medium in the upper wells of Boyden chambers. Upper and lower wells were separated by Matrigel-coated membranes with 8-μm pores. In the lower chambers 2.6 ml culture medium was present with or without HGF/SF (25 ng ml$^{-1}$). After 16 h, all cells that had migrated to the bottom of the lower well were counted with a 20 × ocular of an inverted microscope.

The retroviral packaging Phoenix cells (generous gift from Dr GP Nolan, Stanford University School of Medicine, CA, USA) were transfected using the calcium phosphate transfection system (Gibco-BRL, Breda, The Netherlands) with the c-MET containing vector. Transfected cells were selected by addition of puromycin (2.5 μg ml$^{-1}$; Clontech Laboratories) at day 2. Ten to 14 days after transfection, $6 \times 10^6$ cells were plated per 10-cm petri dish in 10 ml complete medium without pyromycin. The next day, the medium was refreshed and on the following day retroviral supernatant was harvested, centrifuged and frozen (–70°C) in aliquots. Control viral supernatant was collected from cells that had been transfected with the control vector (LZRS-IRES-GFP). Viral supernatants (c-MET-GFP and GFP) were preincubated with dotap (10 μg ml$^{-1}$; Boehringer Mannheim, Mannheim, Germany) on ice for 10 min, and incubated overnight with Ramos cells. After 5 days the transduced Ramos cells were sorted by FACS-STAR on GFP expression (Mann et al, 1983; Kinsella and Nolan, 1996).

### c-MET-staining of transduced cells

Cytospins were prepared of transduced and wild-type Ramos cells, which were fixed with methanol and stained with a polyclonal antibody directed to the last 28 C-terminal amino acids of c-MET (C-28; Santa Cruz; 250 ng ml$^{-1}$) or as a control with a polyclonal control IgG (used at the same concentration as C-28: 250 ng ml$^{-1}$; Southern Biotechnology Associates Inc, Birmingham, AL, USA). Cytospins were washed and incubated with fluorescein isothiocyanate (FITC)-labelled goat anti-rabbit. After washing, cells were examined for c-MET staining with a fluorescent microscope.

### In vitro invasion assay

Boyden chambers (6-well plates; Becton Dickinson) were used to assess tumour cell invasiveness as described previously by Albini et al (1987). Upper and lower chambers of the Boyden chambers were separated by Matrigel-coated filters with 8-μm pores. After rehydration of the Matrigel in IMDM + 0.1% bovine serum albumin (BSA), cells ($5 \times 10^5$) were seeded in the upper chambers in 1.5 ml culture medium (IMDM containing L-glutamin, supplemented with 5% FCS and P/S). HGF/SF (25 ng ml$^{-1}$; produced by COH cells; gift from Prof. T Nakamura, Division of Biochemistry, Biomedical Research Center, Osaka University Medical School, Japan) was added to the lower chambers in 2.6 ml of the same culture medium as present in the upper wells. The chambers were incubated overnight at 37°C. All cells that had migrated from the upper to the bottom of the lower chamber were counted using an inverted light microscope with a 20 × ocular.

### Statistical analysis

Differences in the number of organs of SCID mice invaded by tumour cells between the different groups was determined using the $\chi^2$ test. Differences were considered to be statistically significant at the 5% level. Means ± standard deviation (s.d.) were calculated from proliferation tests.

### RESULTS

### Production of HGF/SF and effect on SCID mice

HGF/SF was produced by Sf9 insect cells using the baculo-gold-virus-producing system. This HGF/SF was used for the in vivo experiments described below. First the biological activity of purified HGF/SF was tested in the presence of FCS to enhance the conversion of pro-HGF/SF into active HGF/SF. HGF/SF promoted the proliferation of NIH3T3-cells, transfected with c-MET, in a dose-dependent manner (Figure 1A). Furthermore, purified HGF/SF promoted colony formation of murine and human haemopoietic progenitor cells. HGF/SF acted synergistic with granulocyte-macrophage colony-stimulating factor (GM-CSF) and interleukin (IL)-3 (Figure 1B). Purified HGF/SF induced scattering of MDCK (Hordijk et al, 1997). The biological activity of the Sf9-derived HGF/SF was similar to recombinant HGF/SF produced by Dr T Nakamura (Figure 1B). The purified HGF/SF from the Sf9 cells was applied for further in vivo studies.

HGF/SF injected s.c. in normal Balb/c mice did not induce marked toxicity in these mice as measured by changes in activity of the mice, haematological cell counts or survival ($n$ = 20; until day 30; results not shown). Also, HGF/SF (5 μg once a day s.c. for 7 days) did not induce toxicity in SCID mice ($n$ = 4; observations for up to 2 months; results not shown).

### Expression of c-Met, HGF/SF production cell lines c-MET expression was determined in the lymphoma cell lines Daudi, Ramos and BJAB using reverse transcription PCR (RT-PCR). c-MET mRNA was detected in the cell line BJAB and not in the cell lines Daudi and Ramos (Weimar et al, 1997). HGF/SF could not be detected (by ELISA) in the supernatant of these three cell lines after 1–3 days of culture (results not shown). The c-MET-negative Ramos cell line was transduced with a retroviral vector, containing either green fluorescence protein (Ramos-GFP) or the c-MET–IRES–GFP (Ramos-c-MET–GFP) sequence. After selection, c-MET-transduced Ramos cells could be identified by both green fluorescence expression as well as by c-MET protein expression (Figures 2 and 3). The biological behaviour of the transfected cells differed from the wild-type Ramos (wtRamos) cells as determined in an in vitro migration assay. In comparison to wtRamos cells or control-transduced (with GFP) Ramos cells, the c-MET–GFP-transduced Ramos cells migrated better in response to HGF/SF (Table 1).

© 1999 Cancer Research Campaign

**Table 2** Effect of HGF/SF on dissemination pattern of BJAB (c-MET+) cells in SCID mice

| Experiment | Organs | Without HGF/SF[a] | With HGF/SFa | |
|---|---|---|---|---|
| 1 | | | (5 μg day$^{-1}$ × 7) | |
| | Lymph nodes[b] | 2/4 | 3/4 | |
| | Kidney | 2/4 | 4/4 | |
| | Lung | 1/4 | 4/4 | |
| | Liver | 0/4 | 0/4 | |
| | Spleen | 0/4 | 0/4 | |
| | Gonads | 0/4 | 0/4 | |
| | Total | 5/24 | 11/24 | |
| 2 | | | (5 μg day$^{-1}$ × 7) | 25 μg day$^{-1}$ × 7) |
| | Lymph nodes[b] | 3/4 | 4/4 | 2/4 |
| | Kidney | 2/4 | 3/4 | 2/4 |
| | Lung | 0/4 | 0/4 | 0/4 |
| | Liver | 0/4 | 0/4 | 0/4 |
| | Spleen | 0/4 | 0/4 | 0/4 |
| | Gonads | 1/4 | 4/4 | 0/4 |
| | Total | 6/24 | 11/24 | 4/24[c] |
| 1+2 | Lymph nodes[b] | 5/8 | 7/8 | |
| | Kidney | 4/8 | 7/8 | |
| | Lung | 1/8 | 4/8 | |
| | Liver | 0/8 | 0/8 | |
| | Spleen | 0/8 | 0/8 | |
| | Gonads | 1/8 | 4/8 | |
| | Total | 11/48 | 22/48[d] | |

Mice were injected i.v. with $5 \times 10^6$ BJAB cells. Either salt or HGF/SF (5 or 25 mg day$^{-1}$) was administered each day s.c. for 7 days to the mice, starting at the day of tumour injection. [a]x/y indicates the number of mice with the indicated phenomenon (x) out of the number of mice in each group (y). [b]The lymph nodes that were involved included: axillary, brachial, poplitial, lumbal, inguinal, caudal and renal; not indicated is the number of lymph nodes in which metastases were formed. [c]Indicates that the number of organs with metastases is significantly decreased ($P$ = 0.029) compared to the number observed with 5 mg HGF/SF per day and is not significantly different ($P$ = 0.0477) from the number observed without HGF/SF. [d]indicates that the number of organs with metastases is significantly different ($P$ = 0.018) from the number obtained without HGF/SF.

These in vitro results suggest that the c-MET–HGF/SF interaction promotes dissemination of lymphoma cells in vivo, like has been observed for transfected NIH3T3 cells and several epithelial cells (Cooper et al, 1986; Stoker et al, 1987; Iyer et al, 1989; Rong et al, 1992, 1993, 1994; Liu et al, 1992; Giordano et al, 1993; Bellusci et al, 1994).

### Effect of HGF/SF on tumorigenicity of c-MET-positive B-lymphoma cells in SCID mice

SCID mice were injected i.v. with $5 \times 10^6$ BJAB cells. HGF/SF was administered s.c. once daily for 7 days. BJAB cells gave tumours in lymph nodes, kidneys, lungs and gonads (Table 2). More mice developed tumours in the different organs when treated with HGF/SF (5 μg per day). Pooling of the six different organs that were investigated of the four mice per group, showed that metastases were formed in 5/24 organs of the mice that were not treated with HGF/SF versus 11/24 organs of the mice that were treated with HGF/SF (mice got ill and were sacrificed 32 days after tumour inoculation) in experiment 1, and that metastases were formed in 6/24 organs of the mice that were not treated with HGF/SF versus 11/24 organs of mice that were treated with HGF/SF (mice got ill and were sacrificed at day 28 and 29) in experiment 2. Adding the results from the two experiments together revealed a significant difference ($P$ = 0.018) in the number of organs with metastases of the mice treated with HGF/SF (5 mg per day) and control mice. In the two experiments, HGF/SF treatment not only promoted dissemination in more mice, but HGF/SF-treated mice also had more lymph node metastases (19 versus eight). Increasing the dose of HGF/SF (from 5 to 25 μg per day × 7 days) did not result in an increased tumorigenicity. Mice treated with 25 μg per day HGF/SF did not have a different dissemination pattern from the control mice (Table 2).

### Effect of HGF/SF on tumorigenicity of c-MET-negative B-lymphoma cells in SCID mice

As a control to prove that HGF/SF increases tumorigenicity of c-MET-positive cells by an effect of HGF/SF on the tumour cells and not on e.g. endothelial or other cells leading to an increased invasion, we studied the effect of administration of HGF/SF on the dissemination pattern of c-MET-negative tumours in SCID mice. The Daudi and Ramos cell lines did not express c-MET as detected by RT-PCR. Mice were injected i.v. with $5 \times 10^6$ tumour cells and 5 μg HGF/SF per day was injected s.c. for 7 days. All mice injected with Daudi cells got sick and were sacrificed at day 32, and mice injected with Ramos cells at day 30. One of the symptoms was paralysis of the hind legs. This symptom was observed in 2/8 mice injected with Daudi cells (one mouse in HGF/SF-treated group and one in the control group) and in 3/8 mice injected with Ramos cells (one mouse in HGF/SF-treated group and two in the control group). Comparing control mice with HGF/SF-treated

*© 1999 Cancer Research Campaign*

**Table 3** Effect of HGF/SF on dissemination of c-MET-negative lymphoma cell lines in SCID mice

| Cell line | Organs | Without HGF/SF[a] | *With HGF/SF[a] |
|---|---|---|---|
| Daudi | Lymph nodes[b] | 3/4 | 1/4 |
| | Kidney | 3/4 | 3/4 |
| | Lung | 0/4 | 0/4 |
| | Liver | 0/4 | 0/4 |
| | Spleen | 0/4 | 0/4 |
| | Ovary | 3/4 | 2/4 |
| | Total | 9/24 | 6/24[c] |
| Ramos | Lymph nodes[b] | 0/4 | 0/4 |
| | Kidney | 2/4 | 0/4 |
| | Lung | 0/4 | 0/4 |
| | Liver | 3/4 | 2/4 |
| | Spleen | 4/4 | 4/4 |
| | Ovary | 1/2 | 1/2 |
| | Total | 10/22 | 7/22[c] |

Mice (all females) were injected i.v. with $5 \times 10^6$ cells. Either salt or HGF/SF (5 μg $day^{-1}$) was administered each day s.c. for 7 days to the mice, starting at the day of tumour injection. Paralysis was present in the hind paws.
[a]x/y indicates the number of mice with the indicated phenomenon (x) out of the number of mice in each group (y). [b]The lymph nodes that were involved included axillary, brachial, poplitial, lumbal, inguinal, caudal and renal; not indicated is the number of lymph nodes in which metastases were formed. [c]Addition of HGF/SF did not result in a significant difference in the number of of mice with metastases in indicated organs: with Daudi-cells: $P = 0.350$; with Ramos-cells: $P = 0.353$.

mice revealed that HGF/SF treatment did not promote dissemination of these two cell lines. There is a tendency that with these cell lines, HGF/SF treatment results in fewer tumours; however, these differences are not significant ($P = 0.350$ for Daudi cells and $P = 0.353$ for Ramos cells; Table 3). These data suggest that HGF/SF did not affect dissemination of lymphoma cells by an indirect effect on e.g. endothelial cells.

### Tumorigenicity after c-MET transduction of c-MET-negative B-lymphoma cells

To strengthen our hypothesis that HGF/SF increased tumorigenicity of c-MET-positive cells, we studied the effect of HGF/SF on tumorigenicity of the c-MET-transduced Ramos cells (Ramos-c-MET), compared to control-transduced cells (Ramos-GFP), which gave promising results in the in vitro assay (Table 1).

Normal HGF/SF (5 μg per mouse per day) and denaturated (30 min autoclaved) HGF/SF were injected s.c. once a day for 7 days. Denaturated HGF/SF was injected instead of salt to exclude the possibility that a low level of endotoxin present in the HGF/SF might influence tumorigenicity of the tumour cells. Denaturated HGF/SF did not have any biological effect in colony assays (data not shown).

Two experiments were performed with four SCID mice per group (Ramos-GFP ± HGF/SF and Ramos-c-MET–GFP ± HGF/SF). Survival did not differ between the four groups; Ramos-GFP (without HGF/SF) 31 ± 1 days, Ramos-GFP (with HGF/SF) 33 ± 2 days, Ramos-c-MET–GFP (without HGF/SF) 29 ± 1 days, and Ramos-c-MET–GFP (with HGF/SF) 32± 2 days. Twelve out of 16 mice were sacrificed when the hind legs became paralysed. In the second experiments almost all mice got sick on day 30 (paralysis 16/16 mice) and all mice were killed on day 31. The results of the two experiments with regard to dissemination are shown in Table 4. c-MET transduction did not result in a different dissemination pattern. HGF/SF treatment seemed to promote dissemination of Ramos-GFP cells, but the increase was not significant ($P = 0.210$). HGF/SF treatment also did not significantly affect dissemination of Ramos-c-MET cells in vivo ($P = 0.142$; Table 4), in contrast to the results of these cells in the in vitro experiments (Table 1).

### DISCUSSION

c-MET is expressed on immature B-cells, such as $CD38^+CD77^+$ tonsillar B-cells and $CD19^+CD20^-$ B-cells, and not on mature B-cells. c-MET expression can be up-regulated by activation of mature B-cells using stimuli such as CD40-ligand, phorbol 12-myristate 13-acetate (PMA) or Epstein–Barr virus (EBV) infection (van der Voort et al, 1997; Weimar et al, 1997). We have previously shown that c-MET is expressed in lymph node samples from non-Hodgkin's lymphoma (NHL) and Hodgkin's disease patients (Weimar et al, 1997). c-MET expression was found in centroblasts of follicular centre cell NHL (8/11 cases were positive), in centroblasts of large B-cell NHL (7/17 cases were positive), and

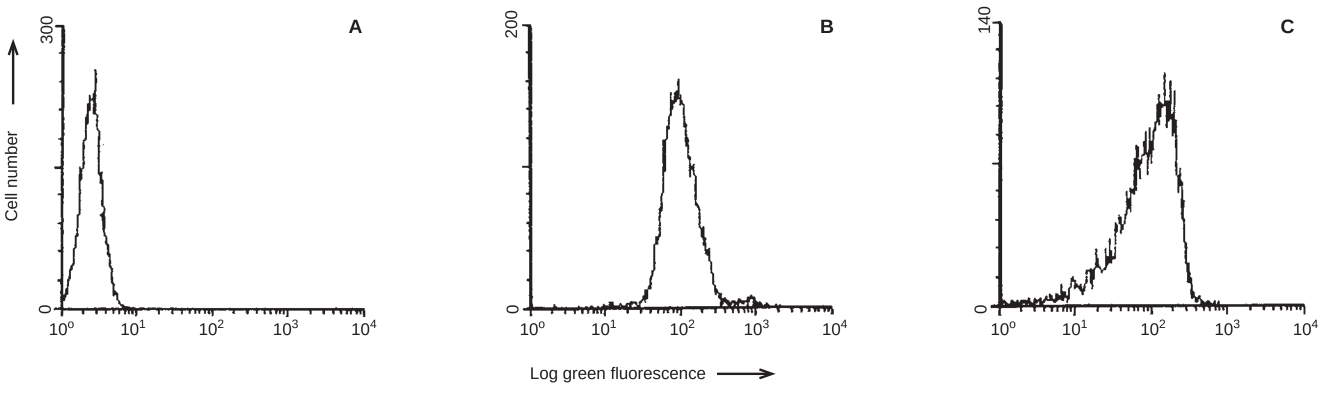

**Figure 2** Flow cytometric analysis of GFP and c-MET–GFP in wild-type (baseline green fluorescence) and transduced (high expression of green fluorescence) Ramos cells. (**A**) Wild-type Ramos cells; (**B**) GFP (control)-transduced Ramos cells; (**C**) c-MET–GFP-transduced Ramos cells

© 1999 Cancer Research Campaign

**Table 4** Tumorigenicity of c-MET transduced Ramos cells in SCID mice

| Organs | Transduction | Without active HGF/SF[a] | With HGF/SF[a] |
|---|---|---|---|
| Lymph nodes[b] | GFP | 2/8 | 2/8 |
| | c-MET/GFP | 3/8 | 1/8 |
| Kidney | GFP | 1/8 | 3/8 |
| | c-MET/GFP | 2/8 | 0/8 |
| Lung | GFP | 0/8 | 0/8 |
| | c-MET/GFP | 0/8 | 0/8 |
| Liver | GFP | 6/8 | 7/8 |
| | c-MET/GFP | 5/8 | 7/8 |
| Spleen | GFP | 7/8 | 8/8 |
| | c-MET/GFP | 8/8 | 5/8 |
| Gonads | GFP | 0/8 | 2/8 |
| | c-MET/GFP | 4/8 | 2/8 |
| Total | GFP | 16/48 | 22/48[c] |
| | c-MET/GFP | 22/48 | 15/48[c] |

Mice (50% females and 50% males per group) were injected i.v. with $5 \times 10^6$ cells. Either inactivated HGF/SF (denaturated) or active HGF/SF (5 μg $day^{-1}$) was administered each day s.c. for 7 days to the mice, starting at the day of tumour injection. Paralysis was present in the hind paws. [a]x/y indicates the number of mice with the indicated phenomenon (x) out of the number of mice in each group (y). [b]The lymph nodes that were involved included: axillary, brachial, poplitial, lumbal, inguinal, caudal and renal; not indicated is the number of lymph nodes in which metastases were formed. [c]Addition of HGF/SF did not result in a significant difference in the number of organs with metastases: with Daudi-cells: $P = 0.350$; with Ramos-cells: $P = 0.353$.

in 1/3 samples of Burkitt's lymphoma. c-MET expression in Hodgkin's disease seemed to be correlated with EBV-expression (c-MET was expressed in 6/8 EBV positive samples, and in none of the 10 EBV-negative samples), which is in concordance with the previously obtained results that EBV can induce c-MET expression.

Several lymphoma cell lines express c-MET (Jücker et al, 1994; van der Voort et al, 1997; Weimar et al, 1997). We found c-MET expression in 2/5 human B-lymphoma cell lines tested (BJAB and Raji were positive; Ramos, Daudi and Jiyoye were negative) (Weimar et al, 1997).

The c-MET ligand HGF/SF is produced by mesenchymal cells, including tonsillar stromal cells and follicular dendritic cells (Van der Voort et al, 1997). For this study we used the c-MET-positive cell line BJAB and the c-MET-negative cell lines Ramos and Daudi for our in vivo experiments, since other investigators have also identified these cell lines as c-MET-positive and -negative respectively (van der Voort et al, 1997; Jücker et al, 1994). We have previously observed that HGF/SF stimulated the migration and invasion of c-MET-positive cell lines, but not of c-MET-negative cell lines (Weimar et al, 1997). In the present study, these results were confirmed. Transduction of Ramos cells with c-MET markedly promoted migration of the transduced cells in response to HGF/SF in an in vitro assay. A response to HGF/SF was only observed when Ramos cells were transduced with c-MET gene (not in control-transduced cells and wild-type cells), suggesting that overexpression of the c-MET gene plays a role in the active migration of lymphoma cells. In a control experiment we determined the effect of HGF/SF on the proliferation of the human B-lymphoma cell lines. None of the cell lines that we have used for our studies (BJAB, Daudi, Ramos and c-MET-transduced Ramos cells) showed an altered proliferation by addition of HGF/SF (excluding the possibility that instead of migration, proliferation had been stimulated).

We could not detect the protein HGF/SF by ELISA in the supernatant of the cell lines. Nakamura et al. (1994) have studied 46 lymphoma B-cell lines and could detect production of HGF/SF in only two B-cell lines. Similar to our studies, the cell lines BJAB, RAMOS and Daudi did not produce HGF/SF. Studies in the normal environment of normal B-cells demonstrated that HGF/SF is produced by stromal cells and suggest that HGF/SF is produced by follicular dendritic cells (van der Voort et al, 1997). These data suggest that any effect of HGF/SF on B-cells is mostly due to paracrine production of HGF/SF in the microenvironment of the tumour. Based on these data and the effect of HGF/SF on migration of B-cells in vitro described above, we started these series of experiments to study the effect of (over)expression of c-MET on the dissemination pattern of human lymphoma cells in SCID mice. Murine and human HGF/SF are highly homologous, and human HGF/SF efficiently activates the murine MET receptor, but murine HGF/SF does not appear to activate efficiently the human MET receptor (Bhargava et al, 1992; Rong et al, 1992, 1994). Therefore, human HGF/SF was exogeneously administered to the mice.

After i.v. administration, liver and kidney are the most active organs sequestering HGF/SF, but detectable levels of radioiodinated HGF/SF could also be recovered from other highly perfused organs such as spleen and adrenals (Appasamy et al, 1993; Zioncheck et al, 1994). The initial half-life of HGF/SF is 3 min and the elimination half-life is approximately 83–114 min. No major toxicities have been reported after i.v. administration of HGF/SF in rats or dogs. The doses applied in this study were equivalent to doses applied in other murine models. In these models, HGF/SF at a dose of 100 μg $kg^{-1}$ protected against renal failure or liver cirrhosis (Matsudo et al, 1995; Ueno et al, 1996; Matsudo et al, 1997).

© 1999 Cancer Research Campaign 

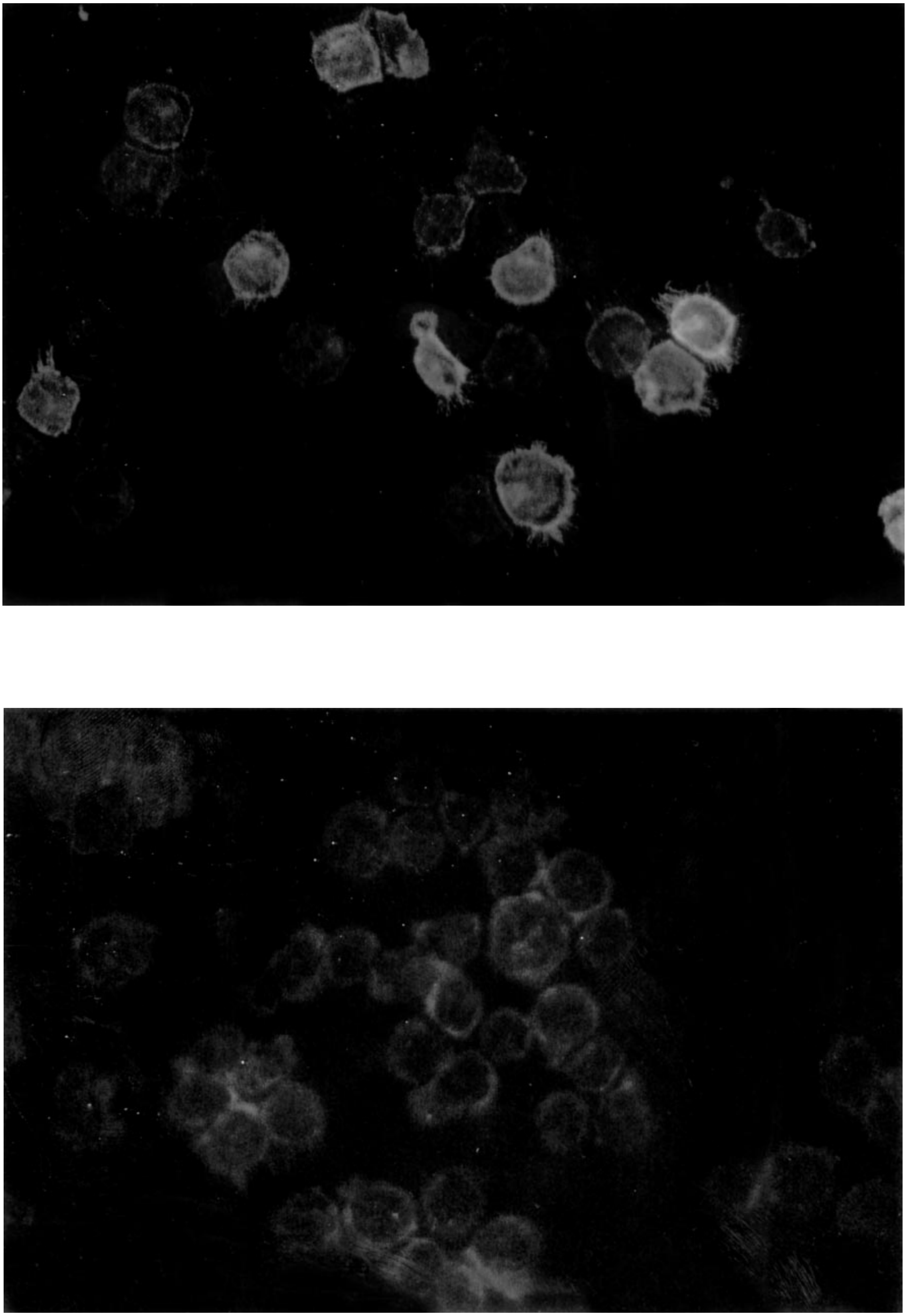

**Figure 3** Expression of c-MET in transduced Ramos B-lymphoma cells: Cytospins of c-MET–GFP transduced Ramos cells (**A**) and, as a control, GFP transduced Ramos cells (**B**) were incubated with a polyclonal (rabbit) antibody directed to the C-terminal part of c-MET (C-28, Santa Cruz), and as a second step with FITC-labelled goat anti-rabbit

 *© 1999 Cancer Research Campaign*

SCID mice have been used succesfully to study dissemination of human lymphoma cells (Cesano et al, 1991; Mule et al, 1992; Walter et al, 1992; de Kroon et al, 1994; Schwartz et al, 1995; Stroeken et al, 1998). Human B-lymphoma cells (e.g. Ramos and Nalm-6) can bind via their $\alpha_4\beta_1$ integins to murine VCAM-1 molecules, because this interaction is not species-specific (Renz et al, 1994; Tsuzuki et al, 1998). Since we have previously observed that HGF/SF induced adhesion of human c-MET-positive B-cells to fibronectin probably via activation of $\alpha_4\beta_1$ integrins (Weimar et al, 1997), we speculated that i.v. injected human c-MET-positive B-cells could adhere to the murine endothelial cells of the SCID-mice via VCAM-1 molecules expressed on these endothelial cells. We hypothesized that addition of human HGF/SF would increase dissemination (via activation of $\alpha_4\beta_1$ integins on the c-MET-positive human B-cells).

In our experiments, exogenous administered HGF/SF significantly promoted the dissemination of (i.v. injected) BJAB cells; more mice developed tumours and more lymph node metastases were scored (Table 2). These results suggested an effect of HGF/SF on dissemination of c-MET-positive BJAB cells. HGF/SF is known to have multiple activities on vascular endothelial cells. Following stimulation of endothelial cell line (ECV304) with HGF/SF, adherence of colon carcinoma cells to the activated endothelial cells increased. Adhesion was mediated by CD44 receptors, since adhesion could be blocked by anti-CD44 antibodies and HGF/SF up-regulated protein expression of CD44 (Hiscox and Jiang, 1997). Furthermore, HGF/SF promotes the growth of endothelial cells (Bussolino, 1992; Nakamura et al, 1996) and motility of endothelial cells (Bussolino, 1992). To exclude the possibility that the observed effects of HGF/SF on the c-MET-positive BJAB cell line was attributable to an effect of HGF/SF on vascular endothelial cells, we also used the c-MET-negative cell lines (Daudi and Ramos). Since HGF/SF did not affect dissemination of these c-MET-negative cell lines, the observed effect of HGF/SF in promoting dissemination of BJAB cells in SCID mice is probably not via an effect on vascular endothelial cells (Table 3).

Overexpression of c-MET has been well documented in various tumours, ranging from melanoma, colectoral carcinoma, thyroid carcinoma, osteosarcoma to leukaemia and lymphoma (Di Renzo et al, 1992, 1995; Liu et al, 1992; Natali et al, 1993; Jücker et al, 1994; Ferracini et al, 1995). The role of overexpression of c-MET on tumorigenicity has been reported for several tumour cell lines. Transfection of murine c-MET in NIH3T3 cells promoted s.c. tumour growth in athymic nude mice, while transfection of human c-MET did not induce tumorigenicity (Rong et al, 1992; Jeffers et al, 1996). The authors explain the differences by the fact that murine HGF/SF does not appropriately stimulate the human c-MET. Co-transfection of human HGF/SF in human c-MET-positive NIH3T3 cells did induce tumorigenicity in 89% of the mice. The same group of investigators have studied the effect of c-MET overexpression in the mouse mammary tumour C127 (Jeffers et al, 1996*a*, 1996*b*). Transfection of neither c-MET nor HGF/SF promoted tumour formation in mice, while co-transfection of both genes markedly induced s.c. tumors. Based on these data we administered human HGF/SF to our mice injected with human c-MET-transduced Ramos cells. In our experiments with c-MET-transduced Ramos cells, c-MET transduction itself did not affect invasion of organs by lymphoma cells. This could be due to the lack of human HGF/SF. However, also the administration of human HGF/SF did not have any effect on tumorigenicity of the c-MET-transduced Ramos cells. HGF/SF administration also did not affect survival times of the mice. Although we can not exclude the possibility that co-transfection of human HGF/SF in these c-MET-transduced cells, creating an autocrine production of HGF/SF, might have different outcomes, our data indicate that the c-MET–HGF/SF interaction only plays a minor role in tumorigenicity of human B-cell lymphoma cell lines in SCID mice.

### ACKNOWLEDGEMENTS

We are very grateful to Eric Notenboom (Division of Cell Biology, Netherlands Cancer Institute) for FACS-sorting the transduced cells, and all people from the Animal Department of the Netherlands Cancer Institute for all their help with the in vivo experiments. This study was supported by the Dutch Cancer Society/KWF, grant 92–57.

### REFERENCES

Adams DH and Lloyd AR (1997) Chemokines: leucocyte recruitment and activation cytokines. *Lancet* **349**: 490–495

Adams DH, Harvath L, Bottaro DP, Interrante R, Catalano G, Tanaka Y, Strain A, Hubscher SG and Shaw S (1994) Hepatocyte growth factor and macrophage inflammatory protein 1β: structurally distinct cytokines that induce rapid cytoskeletal changes and subset-preferential migration in T cells. *Proc Natl Acad Sci USA* **91**: 7144–7148

Albini A, Iwamoto Y, Kleinman HK, Martin GR, Aaronson SA, Kozlowski JM and McEwan RN (1987) A rapid in vitro assay for quantitating the invasive potential of tumor cells. *Cancer Res* **47**: 3239–3245

Appasamy R, Tanabe M, Murase N, Zarnegar R, Venkataramanan R, Van Thiel DH and Michalopoulos GK (1993) Hepatocyte growth factor, blood clearance, organ uptake, and biliary excretion in normal and partially hepatectomized rats. *Lab Invest* **68**: 270–276

Baggiolini M, Dewald D and Moser B (1994) Interleukin-8 and related chemotactic cytokines: CXC and CC chemokines. *Adv Immunol* **55**: 97–179

Bellusci S, Moens G, Gaudino G, Comoglio P, Nakamura T, Thiery J-P and Jouanneau J (1994) Creation of an hepatocyte growth factor/scatter factor autocrine loop in carcinoma cell induces invasive properties associated with increased tumorigenicity. *Oncogene* **9**: 1091–1099

Berman ME, Xie Y and Muller WA (1996) Roles of platelet/endothelial cell adhesion molecule-1 (PECAM-1, CD31) in natural killer cell transendothelial migration and beta 2 integrin activation. *J Immunol* **156**: 1515–1524

Bhargava M, Joseph A, Knesel J, Halaban R, Li Y, Pang S, Goldberg I, Setter E, Donovan MA, Zarnegar R, Micholopoulos GA, Nakamura T, Faletto D and Rosen EM (1992) Scatter factor and hepatocyte growth factor: activities, properties, and mechanism. *Cell Growth Differ* **3**: 11–20

Bussolino F, Di Renzo MF, Ziche M, Bocchietto E, Olivero M, Naldini L, Gaudino G, Tamagnone L, Coffer A, Marchisio PC and Comoglio PM (1992) Hepatocyte growth factor is a potent angiogenic factor which stimulates endothelial cell motility and growth. *J Cell Biol* **119**: 629–641

Butcher EC and Picker LJ (1996) Lymphocyte homing and homeostasis. *Science* **272**: 60–66

Carlos TM and Harlan JM (1994) Leukocyte-endothelial adhesion molecules. *Blood* **84**: 2068–2101

Cesano A, O'Connor R, Lange B, Finan J, Rovera G and Santoli D (1991) Homing and progression patterns of childhood acute lymphoblastic leukemias in severe combined immunodeficient mice. *Blood* **77**: 2463–2474

Cooper CS, Tempest PR, Beckman MP, Heldin CH and Brookees P (1986) Amplification and overexpression of the met gene in spontaneously transformed NIH3T3 mouse fibroblasts. *EMBO J* **5**: 2623–2628

DeGrendele HC, Estess P, Picker LJ and Siegelman MH (1996) CD44 and its ligand hyaluronate mediate rolling under physiological flow: a novel lymphocyte-endothelial cell primary adhesion pathway. *J Exp Med* **183**: 1119–1130

de Kroon JF, Kluin PM, Kluin-Nelemans HC, Willemze R and Falkenburg JH (1994) Homing and antigenetic characterization of a human non-Hogdkin's lymphoma B cell line in severe combined immunodeficient (SCID) mice. *Leukemia* **8**: 1385–1391

Dignass AU, Lynch-Devaney K and Podolsky DK (1994) Hepatocyte growth factor/scatter factor modulates intestinal epithelial cell proliferation and migration. *Biochem Biophys Res Commun* **202**: 701–709

Di Renzo MF, Olivero M, Rerro S, Prat M, Bongarzone I, Pilotti S, Blefiore A, Constantino A, Vigneri R, Pierotti MA and Comoglio PM (1992) Overexpression of the c-MET/HGF receptor gene in human thyroid carcinomas. *Oncogene* **7**: 2549–2553

Di Renzo MF, Olivero M, Giacomini A, Porte H, Chastre E, Mirossay L, Nordlinger B, Bretti S, Bottardi S, Giordano S, Plebani M, Gespach C and Comoglio PM (1995) Overexpression and amplification of the Met/HGF receptor gene during the progression of colorectal cancer. *Clin Cancer Res* **1**: 147–154

Ferracini R, Di Renwo MF, Scotlandi K, Baldini N, Olivero M, Lollini P, Cremona O, Campanacci M and Comoglio PM (1995) The Met/HGF receptor is overexpressed in human osteosarcoma and is activated by either a paracrine or an autocrine circuit. *Oncogene* **10**: 739–749

Galimi F, Bagnara GP, Bonsi L, Cottone E, Follenzi A, Simeone A and Comoglio PM (1994) Hepatocyte growth factor induces proliferation and differentiation of multipotent and erythroid hemopoietic progenitors. *J Cell Biol* **127**: 1743–1754

Gerardi E and Stoker M (1990) Hepatocytes and scatter factor. *Nature* **346**: 228

Giordano S, Di Renzo MF, Narsimhan R, Cooper CS, Rosa C and Comoglio PM (1989) Biosynthesis of the protein encoded by the c-met proto-oncogene. *Oncogene* **4**: 1383–1388

Giordano S, Zhen Z, Medico E, Gaudino G, Galami F and Comoglio PM (1993) Transfer of the motogenic and invasive response to scatter factor/hepatocyte growth factor by transfection of the human c-MET proto-oncogene. *Proc Natl Acad Sci USA* **90**: 649–653

Goff JP, Shields DS, Petersen BE, Zajac VF, Michalopoulos GK and Greenberger JS (1996) Synergistic effects of hepatocyte growth factor on human cord blood $CD34^+$ progenitor cells are the result of c-met receptor expression. *Stem Cells* **14**: 592–602

Goff JP, Shields DS, Boggs SS and Greenberger JS (1997) Effects of recombinant cytokines on colony formation by irradiated human cord blood $CD34^+$ hematopoietic progenitor cells. *Radiat Res* **147**: 61–69

Grant DS, Kleinman HK, Goldber ID, Bhargava MM, Nickoloff BJ, Kinsella JL, Polverini PJ and Rosen EM (1993) Scatter factor induces blood vessel formation in vivo. *Proc Natl Acad Sci USA* **90**: 1937–1941

Heemskerk MH, Blom B, Nolan G, Stegeman AP, Bakker AQ, Weijer K, Res PC and Spits H (1997) Inhibition of T cell and promotion of natural killer cell development by the dominant negative helix loop helix factor Id3. *J Exp Med* **186**: 1597–1602

Hiscox S and Jiang WG (1997) Regulation of endothelial CD44 expression and endothelium–tumour cell interactions by hepatocyte growth factor/scatter factor. *Biochem Biophys Res Commun* **233**: 1–5

Hordijk P, ten Klooster JP, van der Kammen RA, Michiels F, Oomen LCJM and Collard JG (1997) Inhibition of invasion of epithelial cells by Tiam 1-Rac signaling. *Science* **278**: 1464–1466

Iyer A, Kmiecik TE, Park M, Daar I, Blair D, Dunn KJ, Sutrave P, Ihle JN, Bodescot M and Vande Woude GF (1989) Structure, tissue-specific expression, and transforming activity of the mouse met proto-oncogene. *Cell Growth & Differentiation* **1**: 87–95

Jeffers M, Rong S and VandeWoude GF (1996*a*) Hepatocyte growth factor/scatter factor-Met signaling in tumorigenicity and invasion/metastasis. *J Mol Med* **74**: 505–513

Jeffers M, Rong S, Anver M and VandeWoude GF (1996*b*) Autocrine hepatocyte growth factor/scatter factor-Met signaling induces transformation and the invasive/metastatic phenotype in C127 cells. *Oncogene* **13**: 853–856

Jücker M, Günther A, Gradl G, Fonatsch C, Krueger G, Diehl V and Tesch H (1994) The Met/Hepatocyte growth factor receptor (HGFR) gene is overexpressed in some cases of human leukemia and lymphoma. *Leuk Res* **18**: 7–16

Kinsella TM and Nolan GP (1996) Episomal vectors rapidly and stably produce high-titer recombinant retrovirus. *Human Gene Therapy* **7**: 1405–1413

Kmiecik TE, Keller JR, Rosen E and VandeWoude (1992) Hepatocyte growth factor is a synergistic factor for the growth of hematopoietic progenitor cells. *Blood* **80**: 2454–2457

Lawrence MB and Springer TA (1991) Leukocytes roll on a selectin at physiologic flow rates: distinction from and prerequisite for adhesion through integrins. *Cell* **65**: 859–873

Liu C, Park M and Tsa MS (1992) Overexpression of c-met proto-oncogene but not epidermal growth factor receptor or c-erB-2 in primary human colectoral carcinomas. *Oncogene* **7**: 181–185

Mann R, Mulligan RC and Baltimore D (1983) Construction of a retrovirus packaging mutant and its use to produce helper-free defective retrovirus. *Cell* **33**: 153–159

Matsuda Y, Matsumoto K, Ichida T and Nakamura T (1995) Hepatocyte growth factor suppresses the onset of liver cirrhosis and abrogates lethal hepatic dysfunction in rats. *J Biochem* **118**: 643–649

Matsuda Y, Matsumoto K, Yamada A, Ichida T, Asakura HM, Komoriya Y, Nishiyama E and Nakamura T (1997) Preventive and therapeutic effects in rats of hepatocyte growth factor infusion on liver fibrosis/cirrhosis. *Hepatology* **26**: 81–89

Michalopoulos GK (1990) Liver regeneration: molecular mechanisms of growth control. *FASEB* **4**: 176–187

Mizuno K, Higuchi O, Ihle JN and Nakamura T (1993) Hepatocyte growth factor stimulates growth of hematopoietic progenitor cells. *Biochem Biophys Res Commun* **194**: 178–1886

Mule JJ, Jicha DL and Rosenberg SA (1992) The use of congenitally immunodeficient mice to study human tumor metastases and immunotherapy. *J Immunother* **12**: 196–198

Nakamura S, Gohda E, Matsunaga T, Yamamoto I and Minowada J (1994) Production of hepatocyte growth factor by human haematopoietic cell lines. *Cytokine* **6**: 285–294

Nakamura T, Nishizawa T, Hagiya M, Seki T, Shimonishi M, Sugimura A, Tashiro K and Shimizu S (1989) Molecular cloning and expression of human hepatocyte growth factor. *Nature* **342**: 440–443

Nakamura Y, Moritshita R, Higaki J, Kida I, Aoki M, Moriguchi A; Yamada K, Hayashi S, Yo Y, Nakano H, Matsumoto K, Nakamura T and Ogihara T (1996) Hepatocyte growth factor is a novel member of the endothelium-specific growth factors: additive stimulatory effect of hepatocyte growth factor with basic fibroblast growth factor but not with vascular endothelial growth factor. *J Hypertension* **14**: 1067–1072

Naldini L, Vigna E, Ferracini R, Longati P, Gandino L, Prat M and Comoglio PM (1991*a*) The tyrosine kinase encoded by the MET proto-oncogene is activated by autophosphorylation. *Mol Cell Biol* **11**: 1793–1803

Naldini L, Vigna E, Narshiman RP, Gaudino G, Zarnegar R, Michalopoulos G and Comoglio PM (1991*b*) Hepatocyte growth factor (HGF) stimulates the tyrosine kinase activity of the receptor encoded by the proto-oncogene c-MET. *Oncogene* **6**: 501–504

Natali PG, Nicotra MR, Di Renzo MR, Prat M, Bigotti A, Cavaliere R and Comoglio PM (1993) Expression of the c-Met/HGF receptor in human melanocytic neoplasms: demonstration of the relationship to malignant melanoma tumor expression. *Br J Cancer* **68**: 746–750

Nishino T, Hisha H, Nishino N, Adachi M and Ikehara S (1995) Hepatocyte growth factor as a hematopoietic regulator. *Blood* **85**: 3093–3100

Renz ME, Chiu HH, Jones S, Fox J, Kim KJ, Presta LG and Fong S (1994) Structural requirements for adhesion of soluble recombinant murine vascular cell adhesion molecule-1 to $\alpha 4\beta 1$. *J Cell Biol* **125**: 1395–1406

Rong S, Bodescot M, Blair D, Dunn J, Nakamura T, Mizuno K, Park M, Chan A, Aaronson S and Vande Woude GF (1992) Tumorigenicity of the met proto-oncogene and the gene for hepatocyte growth factor. *Mol Cell Biol* **12**: 5152–5158

Rong S, Oskarsson M, Faletto D, Tsarfaty I, Reseau JH, Nakamura T, Rosen E, Hopkins III RF and Vande Woude GF (1993) Tumorigenesis induced by coexpression of human hepatocyte growth factor and the human met protooncogene leads to high levels of expression of the ligand and receptor. *Cell Growth Differ* **4**: 563–569

Rong S, Segal S, Anver M, Reseau JH and Vande Woude GF (1994) Invasiveness and metastasis of NIH 3T3 cells induced by Met-hepatocyte growth factor/scatter factor autocrine stimulation. *Proc Natl Acad Sci USA* **91**: 4731–4735

Rubin JS, Bottaro DP and Aaronson SA (1993) Hepatocyte growth factor/scatter factor and its receptor, the c-met proto-oncogene product. *Biochem Biophys Acta* **1155**: 357–371

Springer TA (1994) Traffic signals for lymphocyte recirculation and leukocyte emigration: the multistep paradigm. *Cell* **76**: 301–314

Stoker M, Gherardi E, Perryman M and Gray J (1987) Scatter factor is a fibroblast-derived modulator of epithelial cell mobility. *Nature* **327**: 239–242

Strieter RM, Standiford TJ, Huffnagle GB, Colletti LM, Lukacs NW and Kunkel SL (1996) ‘The good, the bad and the ugly.’ The role of chemokines in models of human disease. *J Immunol* **516**: 3583–3586

Schwartz MA, Tardelli L, Macosko HD, Sullivan LM, Narula SK and Fine JS (1995) Interleukin 4 retards dissemination of a human B-cell lymphoma in severe combined immunodeficient mice. *Cancer Res* **55**: 3692–3696

Stroeken PJM, Van Rijthoven EA, Van der Valk MA and Roos E (1998) Targeted disruption of the beta-1 integrin gene in a lymphoma cell line greatly reduces metastatic capacity. *Cancer Res* **58**: 1569–1577

Tavassoli M and Hardly C (1990) Molecular basis of homing of intravenously transplanted stem cells to the marrow. *Blood* **76**: 1059–1070

Tsuzusi S, Toyama-Sorimachi N, Kitamura F, Tobita Y and Miyasaka M (1998) FK506 (tacrolimus) inibits extravasation of lymphoid cells by abrogating VLA-4/VCAM-1 mediated transendothelial migration. *FEBS Lett* **430**: 414–418

 *© 1999 Cancer Research Campaign*

Ueno S, Aikou T, Tanabe G, Kobayashi Y, Hamanoue M, Mitsue S, Kawaida K and Nakamura T (1996) Exogenous hepatocyte growth factor markedly stimulates liver regeneration following portal branch ligation in dogs. *Cancer Chemother Pharmacol* **38**: 233–237

van der Voort R, Taher TE, Keehnen RM, Smit L, Groenink M and Pals ST (1997) Paracrine regulation of germinal center B cell adhesion through the c-met-hepatocyte growth factor/scatter factor pathway. *J Exp Med* **185**: 2121–2131

Wakelin MW, Sanz MJ, Albelda SM, Larkin SW, Boughton-Smith N, Williams TJ and Nourshagh S (1996) An anti-platelet-endothelial cell adhesion molecule-1 antibody inhibits leukocyte extravasation from mesenteric microvessels in vivo by blocking the passage through the basement membrane. *J Exp Med* **184**: 229–239

Walter J, Moller P, Moldenhauser G, Schirrmacher V, Pawlita M and Wolf J (1992) Local growth of a Burkitt's lymphoma versus disseminated invasive growth of the autologous EBV-immortalized lymphoblastoid cells and their somatic cell hybrids in SCID mice. *Int J Cancer* **50**: 265–273

Weimar IS, de Jong D, Muller RJ, Nakamura T, van Gorp JMHM, de Gast GC and Gerritsen WR (1997) Hepatocyte growth factor/scatter factor (HGF/SF) promotes adhesion of lymphoma cells to extracellular matrix molecules via $\alpha_4\beta_1$ and $\alpha_5\beta_1$ integrins. *Blood* **89**: 990–1000

Weimar IS, Miranda N, Muller EJ, Kerst JM, de Gast GC and Gerritsen WR (1998) Hepatocyte growth factor/scatter factor (HGF/SF) is produced by human BM stromal cells and promotes proliferation, adhesion and survival of human hemopoietic progenitor cells ($CD34^+$). *Experimental Hematol* **26**: 885–894

Yee CJ, DeFrances MC, Bell A, Bowen W, Petersen B, Michalopoulos GK and Zarnegar R (1993) Expression and characterization of biologically active human hepatocyte growth factor (HGF) by insect cell infected with HGF-recombinant baculovirus. *Biochemistry* **32**: 7922–7931

Zannettino ACW, Berndt MC, Butcher C, Butcher EC, Vadas MA and Simmons PJ (1995) Primitive human hematopoietic progenitors adhere to P-selectin (CD62P). *Blood* **85**: 3466–3477

Zarnegar R and Michalopoulos G (1989) Purification and biological characterization of human hepatopoietin A, a polypeptide growth factor for hepatocytes. *Cancer Res* **49**: 3314–3320

Zarnegar R and Michalopoulos GK (1995) The many faces of hepatocyte growth factor: from hepatopoiesis to hematopoiesis. *J Cell Biol* **129**: 1177–1180

Zioncheck TF, Richardson L, DeGuzman GG, Modi NB, Hansen SE and Godowski PJ (1994) The pharmacokinetic, tissue localization, and metabolic processing of recombinant human hepatocyte growth factor after intravenous administration in rats. *Endocrinology* **134**: 1879–1887

*© 1999 Cancer Research Campaign*
